# Studying on the strictly self-compatibility mechanism of ‘Liuyefeitao’ peach (*Prunus persica* L.)

**DOI:** 10.1371/journal.pone.0200914

**Published:** 2018-08-01

**Authors:** Wei Liu, Maosong Pei, Anning Zhang

**Affiliations:** 1 Shandong Institute of Pomology, Taian, Shandong, People’s Republic of China; 2 College of Horticulture, Nanjing Agricultural University, Nanjing, Jiangsu, People’s Republic of China; Wuhan Botanical Garden, CHINA

## Abstract

Peach (*Prunus persica* L.) generally exhibits self-pollination, however, they can also be pollinated by other varieties of pollen. Here we found two varieties that are different from other peaches: ‘Daifei’ and ‘Liuyefeitao’. ‘Daifei’ produces less pollen, which needs artificial pollination, honeybee pollination, and the fruit setting depends on other varieties of peach pollen. ‘Liuyefeitao’ exhibits strictly self-pollination, hence pollen from other species is rejected. To explore the mechanism of this phenomenon, we performed a high-throughput sequencing of the stigma (including style) of ‘Daifei’ and ‘Liuyefeitao’ to explain the rejection mechanism of other varieties of pollen of ‘Liuyefeitao’ peach. In our study, we found one S gene, and lots of non-S-locus factors such as: F-box proteins, Ub/26S, MAPKs, RLK, and transcription factor were differential expressed between ‘Daifei’ and ‘Liuyefeitao’. We supposed that the strictly self-compatible of ‘Liuyefeitao’ may result from the synthesis of these factors.

## Introduction

There are two types of self-incompatibility (SI) in plants: sporophytic self-incompatibility (SSI) and gametophytic self-incompatibility (GSI) according to how the incompatibility phenotype of the pollen grain is determined. The SSI type plants include the Asteraceae, Cruciferae and Convolvulaceae families, etc. [1]. The GSI type plants included the Papaveraceae, Scrophulariaceae, Gramineae, Solanaceae, and Rosaceae families, etc. [1]. In the GSI reaction, two different mechanisms were mainly studied, one is the Ca^2+^-mediated signaling cascade in the pollen tube cytoplasm in Papaveraceae; the other is based on *S*-nuclease in Scrophulariaceae, Gramineae, Solanaceae, and Rosaceae. Studies predicted that the *S-RNase* in the stigma and *SFB* in the pollen plays an important role in the GSI in S-nuclease-based SI system [2]. When pollinated by the same *S* genotype pollen, S-RNase specifically disrupted tip-localized reactive oxygen species (ROS) of incompatible pollen tube. Then the disruption of tip-localized ROS decreased the Ca^2+^ current, depolymerized the actin cytoskeleton, and degraded the nuclear DNA. It indicated that programmed cell death (PCD) may occur in SI response [3–6].

The study of S-RNase has a long history. Lewis firstly isolated protein related to specific *S* allele in *Oenothera organesis* in 1952. Then Bredemeijer and Blass isolated a pistil glycoprotein linked to the *S*-allele in *Nicotiana alata* in 1981 [7]. S-RNase is a secreted glycoprotein in the pistil and the concentration of S-RNase is highest in the upper 1/3 of the style where the SI pollen tube growth is strongly inhibited [8,9]. The accumulation of S-RNase is consistent with the development of flowers, which means that the expression of S-protein and *S*-mRNA were both very low in the bud stages, but significantly increased at the early bloom stages. The SI buds can accept self-pollen in the immature period but repel self-pollen as S-RNase content increases in the early bloom stages. This phenomenon suggested that the conversion from SI to (self-compatibility) SC requires the accumulation of a certain amount of S-protein and *S*-mRNA [10, 11]. The *S* genes in the pollen belong to a class of *SFB* (S-locus-encoded F-box genes) gene. The F-box protein exists as a component of the ubiquitin ligase SCF complex. There is a very important regulatory approach in plant cells, which is to degrade the protein by the SCF complex-mediated ubiquitin/26S protein body. In this process, the F-box protein acts as a receptor, recruiting the target protein and ligate to the core complex for ubiquitination [12]. The type and distribution of polypeptide signals in the extracellular matrix of pistil, can affect the microfilament skeleton, the cellular exocytosis, the flow of cytoplasm, the concentration gradient of calcium ion and the growth direction of the pollen tube, such as LAT52, LeSTIG1, and TTS [13–15]. Along with the same function of mitogen-activated protein kinases (MAPK) such as LePRKs, P26.1, and P56 in the Pollen tube [16–18].

Peach (*Prunus persica* L.) generally exhibits self-pollination, however, they also can be pollinated by other varieties of pollen. Here we found two varieties that are different from other peaches: ‘Daifei’ and ‘Liuyefeitao’. ‘Daifei’ produces less pollen, which needs artificial pollination, honeybee pollination, and the fruit setting depends on other varieties of peach pollen. ‘Liuyefeitao’ exhibits strictly self-pollination, hence pollen from other species is rejected (After the artificial emasculation, pollination with other varieties of pollen does not yield fruit, only their own pollen pollination can produce fruit). We performed a high-throughput sequencing of the stigma (including style) of ‘Daifei’ and ‘Liuyefeitao’ to explain the rejection mechanism of other varieties of pollen of ‘Liuyefeitao’ peach.

## Materials and methods

### Plant material

The stigma and style from ‘Daifei’ and ‘Liuyefeitao’ flowers were collected at the early bloom stages in the peach garden of Tianping lake experimental bases of Shandong fruit research institute in spring 2017. The flowers were immediately frozen in liquid N and stored at -80°C. Three biological replicates were used. To test the self- and cross-(in)compatibility of styles of ‘Liuyefeitao’, the fruit setting rate of ‘Liuyefeitao’ and ‘Daifei’ was calculated when pollinated by the pollen of ‘Zhonghuashoutao’, ‘Hanlumi’, ‘Chaohong’, and ‘Liuyefeitao’ in the peach garden of Tianping lake experimental bases of Shandong fruit research institute in 2016.

### RNA library preparation and sequencing

Total RNA was obtained from the flowers samples using RNAprep Pure Plant Kit (Polysaccharides&Polyphenolics-rich) (Tiangen Biotech, Beijing, China) following the manufacturer’s procedure. The RNA concentration and purity were checked by OD A260/A280 (>1.8) and A260/A230 (>1.6), and the yield and quality were accessed using Agilent 2100 Bioanalyzer (Agilent Technologies, Santa Clara, CA, USA) and RNA 6000 Nano LabChip Kit (Agilent, CA, USA), RIN>7.0. The preparation of whole transcriptome libraries and deep sequencing were performed by Beijing Ori-Gene Science and Technology CoRP., LTD (Beijing, PR China). Transcriptome libraries were constructed using NEBNext^®^ Ultra^™^ RNA Library Prep Kit for Illumina (New England Biolabs) according to the manufacturer’s instructions. Libraries were controlled for quality and quantified using the BioAnalyzer 2100 system and qPCR (Kapa Biosystems, Woburn, MA, USA). The resulting libraries were sequenced initially on a HiSeq2000/2500 instrument that generated paired-end reads of 100/150 nucleotides.

### Data processing

Raw sequencing reads were checked for potential sequencing issues and contaminants using FastQC. Adapter sequences, primers, Ns, and reads with quality scores below 30 were trimmed. Reads with a length < 60 bp after trimming were discarded. Sequence reads were aligned to the prunus persica genome (ftp://ftp.ensemblgenomes.org/pub/plants/release-35/fasta/prunus_persica/dna/Prunus_persica.Prupe1_0.dna.toplevel.fa.gz) using the TopHat 2.0 program [19], and the resulting alignment files were reconstructed with Cufflinks. The transcriptome of each sample was assembled separately using Cufflinks 2.0 program. All transcriptomes were pooled and merged to generate a final transcriptome using Cuffmerge. After the final transcriptome was produced, Cuffdiff was used to estimate the abundance of all transcripts based on the final transcriptome. For mRNA analyses, the RefSeq and Ensembl transcript databases were chosen as the annotation references. We used the Potential Calculator (CPC) [20] to predict transcripts with coding potential. The read counts of each transcript were normalized to the length of the individual transcript and to the total mapped read counts in each sample and expressed as fragments per kilobase of exon per million mapped reads (FPKM). Sequence reads mapped to different isoforms of individual genes were pooled together for subsequent comparative analyses.

In analysis, a criterion of |log2(fold-change)|≥1 and P value≤0.05 between the two conditions was used to identify differentially expressed genes (DEG). DEG were set as the foreground and all of the transcripts as the background, Hyper-geometric distribution was employed to detect the significant GO terms and KEGG pathways at a significance level of 0.05.

### RT-PCR analysis

Ten genes from the DE genes of ‘Daifei’ and ‘Liuyefeitao’ were selected randomly. Total RNA was separately extracted from stigma and style of ‘Daifei’ and ‘Liuyefeitao’ at early bloom stages using Plant Total RNA Isolation Kit Plus (FOREGENE, Chengdu, China). Gene-specific primers were designed according to the reference sequences using Primer Premier 5.0 (S1 Table). The first strand of cDNA, synthesized with reverse transcription following the TransScript One-Step gDNA Removal and cDNA Synthesis SuperMix Kit’s instructions (TransGen, Beijing, China), was used as template for qRT-PCR to detect the expression level of candidate genes. Real-time quantification was performed on the Lightcycle-480 (Roche) and the LightCycler 480 SYBR Green I Master (Roche). The Expression levels of candidate genes were calculated by using the 2-ΔΔCt method. All PCR experiments were performed on three independent biological samples, each including three technical replicates. The peach actin gene 18S rRNA was used as a normalizer.

## Results

### The data of fruit setting rate in self- and cross-fertilization of ‘Daifei’ and ‘Liuyefeitao’

To test the self- and cross-(in)compatibility of styles of ‘Liuyefeitao’, the pollen that derive from ‘Zhonghuashoutao’, ‘Hanlumi’, ‘Chaohong’, and ‘Liuyefeitao’ were used as pollinator. As a result, the fruit set of self-pollination in ‘Liuyefeitao’ is 7.72%, while none fruit is found from cross-pollination (Table 1). In contrast, all the four pollinators are cross-compatible with the styles of ‘Daifei’. Thus, ‘Liuyefeitao’ is a strictly self-pollination cultivar.

**Table 1:** Fruit setting rate in self- and cross-fertilization of ‘Daifei’ and ‘Liuyefeitao’.

Male parent	Female parent	Flower number	Fruit number	Fruit set (%)
Zhonghuashoutao	Liuyefeitao	1826	0	0
Hanlumi	Liuyefeitao	1408	0	0
Chaohong	Liuyefeitao	1684	0	0
Liuyefeitao	Liuyefeitao	2047	158	7.72
Zhonghuashoutao	Daifei	1069	268	25.07
Hanlumi	Daifei	936	209	22.32
Chaohong	Daifei	893	224	25.08
Liuyefeitao	Daifei	1362	173	12.7

### Transcriptome sequencing and assembly

Illumina sequencing data from ‘Daifei’ and ‘Liuyefeitao’ peach was deposited in the NCBI SRA database under accession number SRP149753, bioProject accession: PRJNA474565. In total 483.777 M raw reads were generated by Illumina sequencing of ‘Daifei’ and ‘Liuyefeitao’. There were 449.091 M clean reads after removing low-quality sequences (Table 2). We used Tophat2 [21] to map clean data to reference genes. The mapping rate refers to the percentage of mapped reads in clean reads, which most directly reflected the utilization rate of sequencing data. In addition to the quality of sequencing, the mapping rate is related to the completeness of reference genome assembly and annotation. It is also related to the genomic relationship and sample treatment. We can assess whether the sequencing results and selected reference genomes meet the needs of information analysis through the mapping rate. The mapping rate of ‘Daifei’ and ‘Liuyefeitao’ to reference genome was list in Table 3. The result showed that the minimum ‘total mapping rate’ of ‘Daifei’ and ‘Liuyefeitao’ was 86.24% and 89.23% respectively suggesting the validity of our data.

**Table 2:** Summary of the sequence analyses.

Sample	Raw Reads(M)	Raw Bases(G)	Raw Q20(G)	Raw Q30(G)	Clean Reads(M)	Clean Bases(G)	Clean Q20(G)	Clean Q30(G)	Average Length(bp)
A1	41.017	6.153	5.939 (96.5%)	5.663 (92.0%)	38.148 (93.0%)	5.608 (91.1%)	5.511 (98.3%)	5.333 (95.1%)	147
A2	44.567	6.685	6.436 (96.3%)	6.119 (91.5%)	41.330 (92.7%)	6.061 (90.7%)	5.950 (98.2%)	5.749 (94.9%)	146.6
A3	36.31	5.447	5.261 (96.6%)	5.021 (92.2%)	33.937 (93.5%)	4.980 (91.4%)	4.896 (98.3%)	4.739 (95.2%)	146.8
C1	34.629	5.194	5.000 (96.3%)	4.760 (91.6%)	32.043 (92.5%)	4.696 (90.4%)	4.612 (98.2%)	4.461 (95.0%)	146.5
C2	46.005	6.901	6.653 (96.4%)	6.338 (91.9%)	42.650 (92.7%)	6.264 (90.8%)	6.155 (98.2%)	5.954 (95.0%)	146.9
C3	37.698	5.655	5.436 (96.1%)	5.168 (91.4%)	34.772 (92.2%)	5.097 (90.1%)	5.004 (98.2%)	4.837 (94.9%)	146.6

The statistics are the result of read1+read2.

A, ‘Daifei’; C, ‘Liuyefeitao’.

Raw Data, raw Data statistics, sequence reads and bases of each sample.

Clean Data, valid data, the number and proportion of reads and bases of each sample after quality pretreatment.

Q20 and Q30, the percentage of bases with Phred values >20 and >30, respectively.

**Table 3:** Statistics of mapping ratio to reference genome.

Sample	Total Reads(M)	Total Mapped(M)	Multiple Mapped(M)	Uniquely Mapped(M)
A1	38.148	33.388(87.52%)	0.968(2.54%)	32.420(84.98%)
A2	41.33	35.724(86.44%)	1.166(2.82%)	34.558(83.61%)
A3	33.937	29.267(86.24%)	0.803(2.37%)	28.464(83.87%)
C1	32.043	28.644(89.39%)	1.079(3.37%)	27.565(86.03%)
C2	42.65	38.746(90.85%)	0.980(2.30%)	37.767(88.55%)
C3	34.772	31.029(89.23%)	1.226(3.53%)	29.803(85.71%)

A, ‘Daifei’; C, ‘Liuyefeitao’.

Total Reads, total reads for comparison; Total Mapped, the number and proportion of reads mapped to reference genome; Multiple Mapped, mapped reads of multiple alignment positions. Uniquely Mapped, mapped reads of only one position.

### Gene expression analysis

**Fig 1:**
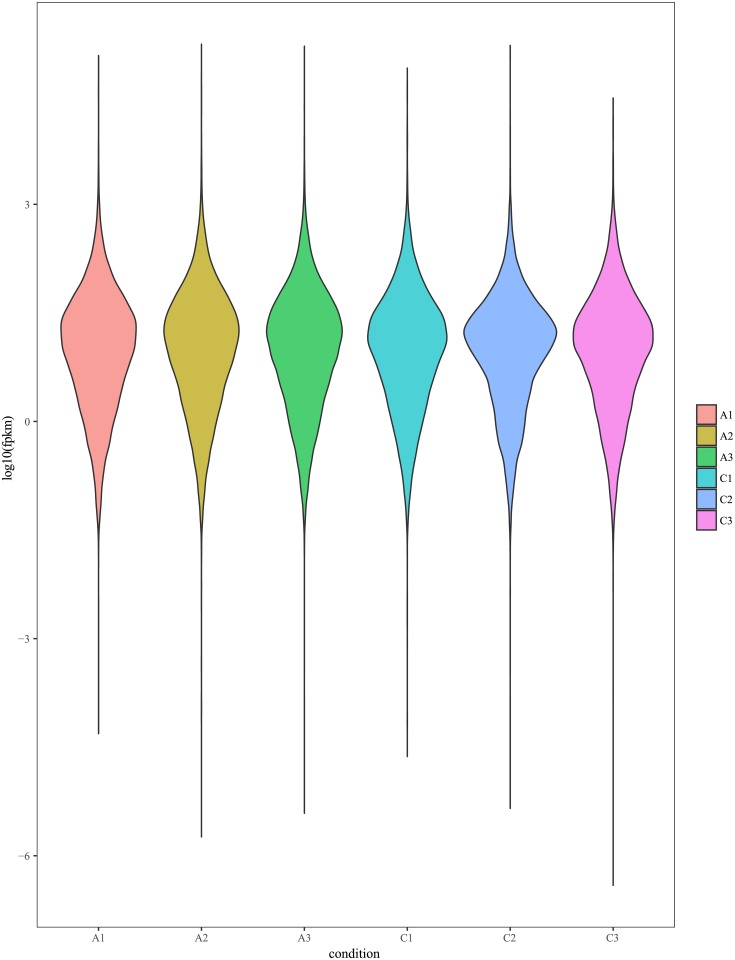
Gene expression distribution. A, ‘Daifei’; C, ‘Liuyefeitao’. The Y-axis is log_10_(fpkm), the X-axis are three biological repetitions of ‘Daifei’ and ‘Liuyefeitao’ respectively.

**Fig 2:**
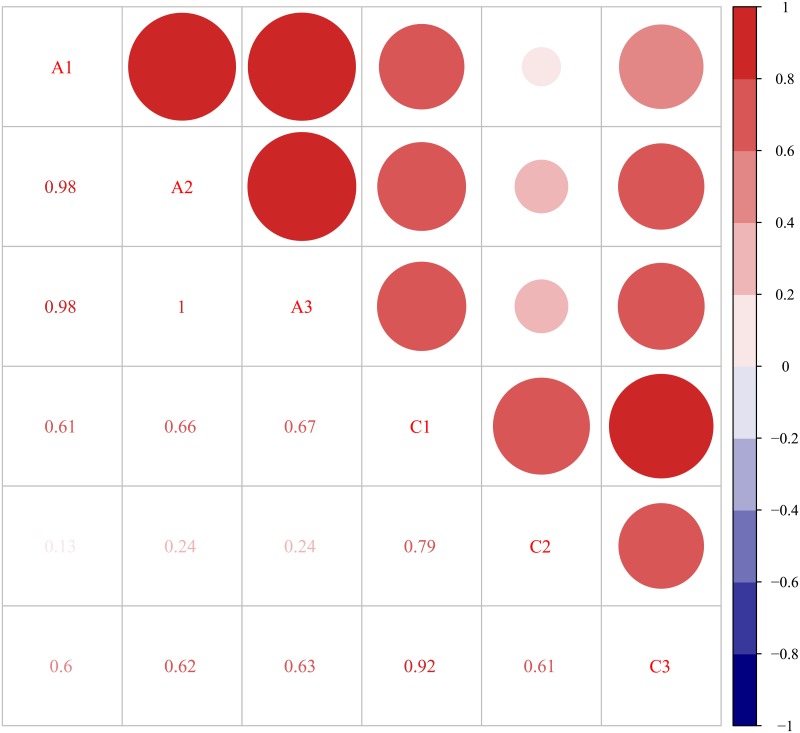
Correlation analysis between samples. A, ‘Daifei’; C, ‘Liuyefeitao’. The bottom left of the graph is the correlation coefficient, the higher the correlation coefficient is, the darker and larger the circle at the upper right of the graph is.

We used FPKM (Fragments Per Kilobase of transcript per Million fragments mapped) to measure the abundance of transcription or gene [22]. The distribution of gene in each sample was shown in Table 4 and Fig 1. For biological replicate samples, it is important to assess the relevance of biological replication for the analysis of transcriptome sequencing results. Biological repeated correlation can not only test the repeatability of biological experiments, but also evaluate the reliability of differentially expressed genes and screen abnormal samples. Pearson's correlation coefficient is an evaluation index of biological repeated correlation. The closer the r is to 1, the stronger the correlation between the two repeated samples. The correlation between samples was shown in Fig 2.

**Table 4:** Distribution interval statistics of gene expression in each group.

Sample	0–0.5	0.5–1.0	1.0–5.0	5.0–10.0	10.0–50.0	>50.0
Daifei	1612(8.55%)	1091(5.79%)	3960(21.01%)	2480(13.16%)	6457(34.25%)	3252(17.25%)
Liuyefeitao	1445(7.54%)	1005(5.24%)	3838(20.03%)	2712(14.15%)	7213(37.63%)	2953(15.41%)

### Differential expression analysis

**Fig 3:**
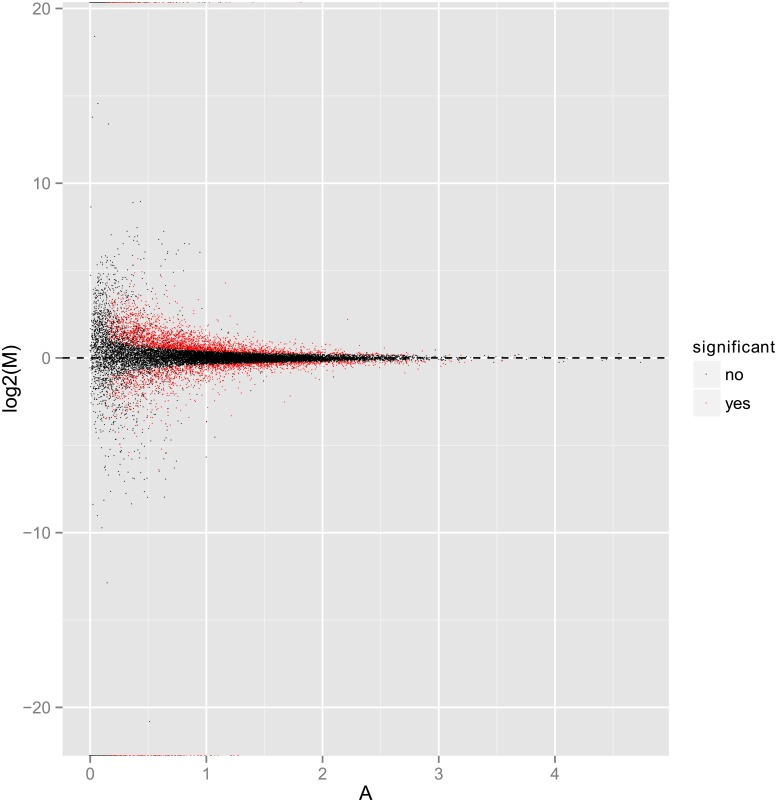
The volcano map of differentially expressed gene. The red circles represent DEGs in ‘Liuyefeitao’ compared to ‘Daifei’.

Gene or transcript expression has temporal and spatial specificity. External stimuli and internal environment are both factors that affect the expression of genes or transcripts. Genes with significant differences in expression levels under different conditions are known as differentially expressed genes. We made differential expression of ‘Daifei’_VS_‘Liuyefeitao’. In the paper, up-regulated genes were referred as the gene expression level was greater in samples ‘Liuyefeitao’ than in ‘Daifei’. Down-regulated genes means the gene expression level was less in ‘Liuyefeitao’ than in ‘Daifei’. In total, 4206 differentially expressed genes (DEG) were obtained from the 19379 genes. Among all the DEGs, there were 2658 DEGs up-regulated and 1548 DEGs down-regulated. The volcano plot visually shows the relationship between the level of significance and the fold change. The volcano map of differentially expressed gene was shown in Fig 3.

### Gene function annotation

**Fig 4:**
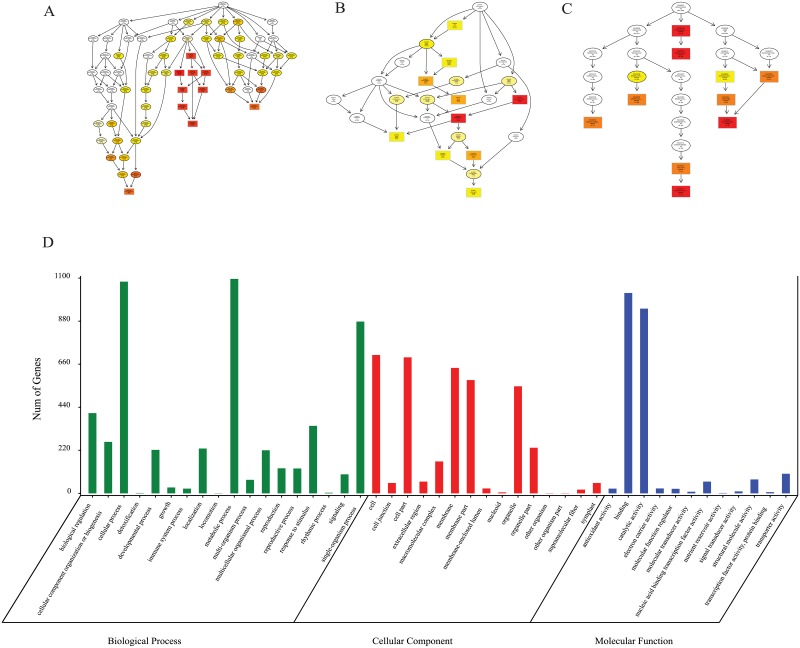
GO analysis of DEGs. Directed Acyclic Graph (DAG) is the graphical display of GO enrichment results with candidate targeted genes (A-C). The number of genes in GO term was showed in histography (D).

Gene Ontology (GO) is an internationally standardized classification system of gene functions, which provides a dynamically updated standard vocabulary to fully describe the properties of genes and gene products in organisms. The GO has three ontologies (ontology) that describe the molecular function of the gene, the cellular component, and biological process. Directed acyclic graph (DAG) is a graphical display of DE genes GO enrichment analysis results. The branch represents the relationship of inclusion, which defines the scope from increasingly small from top to bottom. The top 10 results of GO enrichment analysis are selected as the master node of DAG, and are shown together related by GO term by including the relationship, and systematically GO term shown together, where the color depth represents the enrichment degree. DAGs were drawn in the biological process (BP), cellular component (CC) and molecular function (MF), respectively. The DAG of BP, CC and MF of DEGs were shown in Fig 4. The results showed that 11305 DEGs were enriched in 45 GO terms. Genes enriched in ‘immune system process’, ‘localization’, ‘reproduction’, ‘reproductive process’, ‘response to stimulus’, ‘signaling’, ‘antioxidant activity’ and ‘signal transducer activity’ may involve in pollen recognition.

**Fig 5:**
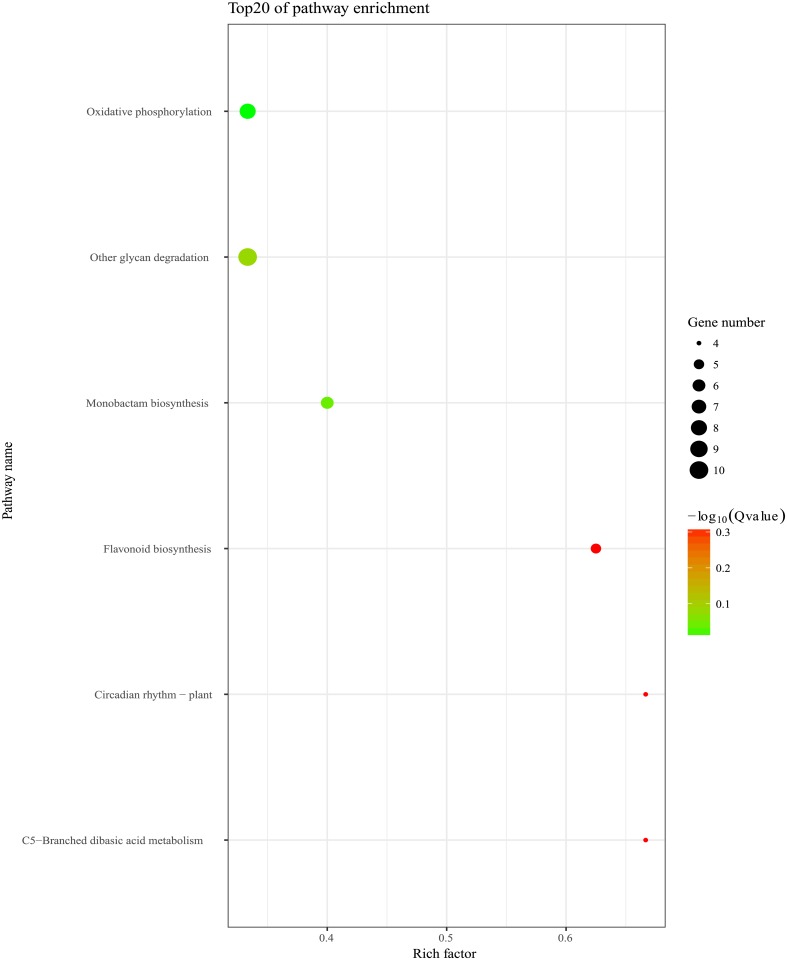
KEGG analysis of DEGs. The area of the circle represents the number of genes, the larger the area of the circle, the greater the number of genes. The color of the circle indicates the degree of enrichment, the higher the degree of enrichment, the more red.

In *vivo*, different genes coordinate with each other to exercise their biological functions, based on the pathway analysis helping to further understand the biological functions of genes. We also made KEGG enrichment analysis of the metabolic pathways of the DEGs. In total, 37 DEGs were enriched in 6 KEGG pathways. The top three KEGG pathways were ‘Other glycan degradation’, ‘Oxidative phosphorylation’, ‘Monobactam biosynthesis’ (Fig 5).

To obtain more gene annotation information, we also performed BLAST of DEGs against the NCBI non-redundant (Nr) database. The result showed that 257 DEGs related to pollen recognition can be divided into five clusters such as: S-RNase, F-box protein, ubiquitin, mitogen-activated protein kinases (MAPKs) signaling pathway, and receptor-like kinase (RLK). Moreover190 transcription factor were also identified (S2 Table).

### Real-time quantitative PCR

**Fig 6:**
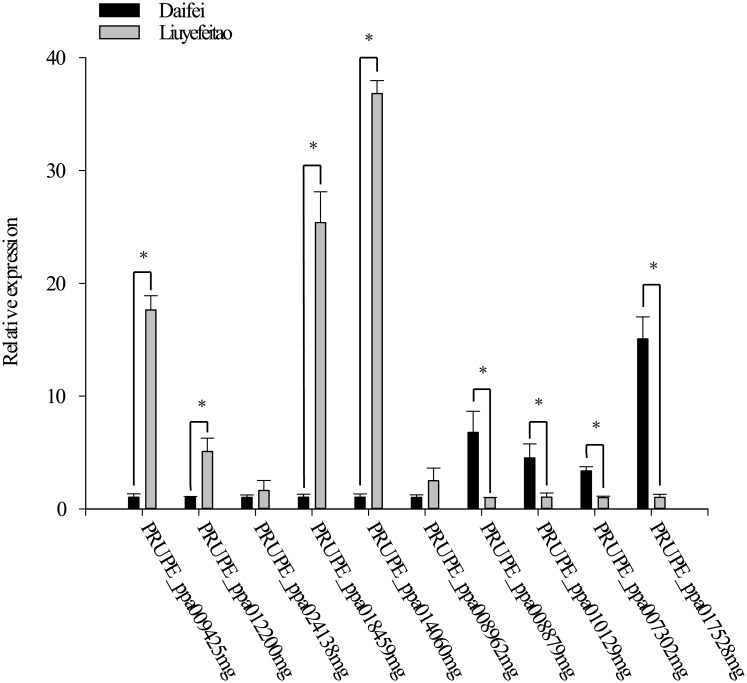
The expression of ten selected DEGs. The X-axis represents the gene name, and the Y-axis represents the relative expression of gene. ‘*’indicate significant differences (P < 0.01).

We randomly selected ten genes from the DEGs for real-time quantitative PCR. These DEGs included two MAPK signaling pathway genes ‘p90 ribosomal S6 kinase’ (PRUPE_ppa009425mg) and ‘serine/threonine-protein phosphatase 2B catalytic subunit’ (PRUPE_ppa007302mg), two ubiquitin related genes ‘ubiquitin-conjugating enzyme (E2)’ (PRUPE_ppa012200mg) and ‘E3 ubiquitin-protein ligase RMA1H1’ (PRUPE_ppa010129mg), two F-box genes ‘F-box/kelch-repeat protein’ (PRUPE_ppa024138mg) and ‘F-box protein PP2-B12’ (PRUPE_ppa008879mg), one ‘S-RNase’ (PRUPE_ppa018459mg), one ‘transcription factor RADIALIS’ (PRUPE_ppa014060mg) and one ‘mitochondrial import receptor subunit TOM40-1’ (PRUPE_ppa008962mg). The gene expression was shown in Fig 6. The total expression pattern of the ten genes obtained with qRT-PCR was consistent with the RNA-seq data. The RT-PCR result confirmed the validity of our high-throughput sequencing results.

## Discussion

The S-RNase play an important role in GSI, which is necessary and sufficient for pollen recognition [23]. Lots of S-RNase have been isolated in Rosaceae, such as: *Malus pumila* [24], *Pyrus serotina* [25], *Prunus avium* [26], *Prunus dulcis* [27], *Prunus salicina* [28], *Eriobotrya japonica* [29] and *Prunus armeniaca* [30]. We found a highly expressed gene (PRUPE_ppa018459mg) in ‘Liuyefeitao’ which is homologous to the *S-RNase* gene of *Prunus dulcis* (S2 Table). The FPKM of PRUPE_ppa018459mg in ‘Liuyefeitao’ (4507.88) is 900 times that of ‘Daifei’ (5.20109), suggesting that this gene is closely related to recognition of pollen of ‘Liuyefeitao’.

F-box protein is one of the largest family in plant [31], which play an important role in protein ubiquitination and degradation [32]. Studies have proved that F-box proteins regulated numerous biological processes, such as lateral root development [33], hormonal responses [34], and senescence [35] and so on. F-box proteins have various protein-protein interaction domains at their C terminus, such as LRR, kelch repeats, FBD, WD40, PAS/PAC, ring finger, tubby (TUB), and PPR in *Arabidopsis* [36, 37]. In this study, we found three F-box/LRR-repeat protein, five F-box/kelch-repeat protein, and twelve F-box proteins were up-regulated in ‘Liuyefeitao’ compared to ‘Daifei’ (S2 Table). We also found five F-box/LRR-repeat protein, seven F-box/kelch-repeat protein, and five F-box protein down-regulated in ‘Liuyefeitao’ compared to ‘Daifei’ (S2 Table). The self-incompatible process is a pollen and stigma interactions process, which specifically identify their own pollen [38], and these F-box proteins may play an important role in pollen recognition.

Selective degradation of proteins effectively regulates the turnover of functional proteins. It is one of the important links of precise regulation of life process. The ubiquitination and 26S proteosome system (Ub/26S) is one of the most important, highly selective protein degradation pathway which regulate various metabolic processes such as: plant pollen germination, pollen tube elongation and SI response [39]. The ubiquitin-activating enzyme E1 (UBE1), ubiquitin conjugation enzyme E2 (UBE2), and ubiquitin ligase E3 (UBE3) are three key enzymes involved in the Ub/26S system [40]. The U-box protein was one of the three families of E3 ligase, the other two families were RING and HECT protein [41, 42]. The S-phase kinase-associated protein1 (SKP1) was one of the most important elements of E3 ligase: SKP1/Cullin/F-box (SCF) complex [43]. Currently, many studies have shown that inhibition of ubiquitin-proteasome activity significantly decreases pollen tube growth and alters pollen tube morphology in *Actinidia deliciosa* [44], *Antirrhinum* [45], and *Picea wilsonii* [46]. We found lots of genes related to ubiquitin differential expressed, such as U-box domain-containing protein, SKP1-like protein, E3 ubiquitin-protein ligase, and so on (S2 Table). These ubiquitin proteins may involve in Ub/26S proteasome pathway to degrade the non-self S-RNase of ‘Liuyefeitao’ peach.

Mitogen-activated protein kinases (MAPKs) formed highly conserved signaling networks in eukaryotic cells’ signal perception and signal transduction [47]. They have been shown to be activated by a variety of stresses, including wounding, drought, cold, heat, UV, touch, osmotic shock, and salt [48–50]. The recognition of pollen is a strictly events regulating pollination and fertilization and involve many signaling pathway. Studies have found that MAP Kinase contributes to the self-incompatibility response in *Papaver somniferum* [47, 51]. When compared the DEGs between ‘Daifei’ and ‘Liuyefeitao’, we found lots of MAPKs genes differential expressed, including ‘p90 ribosomal S6 kinase’ and ‘serine/threonine-protein phosphatase 2B catalytic subunit protein kinases’ (S2 Table). These MAPKs gene may take part in the pollen-stigma interaction signaling events and regulate pollination and fertilization.

Protein kinase (PKs) is a big family of receptor-like kinase (RLK), which play important role in development, self-incompatibility, male sterile, stress resistance, disease resistance etc. [52, 53]. The Leucinerich repeat receptor-like kinase (LRR-RLK) gene is another big family of RLK, which has been isolated in numerous plants [54, 55]. A typical LRR-RLK contains multiple extracellular leucine-rich repeat domain, a single transmembrane domain, and an intracellular kinase domain. LRR-RLKs activate autophosphorylation of intracellular kinase domain by specific binding to ligands, converting extracellular signals to cytoplasmic signals, and thus affecting plant growth and development [53, 56]. We found many leucine-rich repeat receptor protein kinase, and receptor-like protein kinase differential expressed in our experiment (S2 Table). These leucine-rich repeat receptor protein kinases may play an important role in transmembrane transmission of pollen recognition signals.

Transcription factors (TFs) can bind to a specific gene sequence upstream and regulate gene expression in a time-specific and tissue-specific manner [57, 58]. As one of the most numerous transcription factors in plants, the members of AP2/ERF superfamily were reported extensively to be involved both in the regulation of the process of growth and development in plant [59]. The WRKY family is a superfamily of TFs, which is associated with both senescence and defense related processes, and plant development [60, 61]. ERF transcription factors are important regulatory components of ethylene signaling, known to be involved in plant development and stress responses by regulating the expression of ethylene responsive genes [62]. To date, few studies about transcription factor involved in pollen recognition have been identified. In this study, we found lots of transcription factor differential expressed between ‘Daifei’ and ‘Liuyefeitao’, such as ‘AP2-like ethylene-responsive transcription factor’, ‘WRKY transcription factor’, ‘MADS-box protein’, ‘transcription factor bHLH’, ‘MYB’ and so on (S2 Table).

## Conclusion

In the natural state, peach generally exhibits self-pollination, but can accept pollen from other varieties. Only the ‘Liuyefeitao’ is different from other peaches, which can only self-pollination. To explore the mechanism of this phenomenon, we selected ‘Daifei’ which produces less pollen, and the fruit setting depends on other varieties’ pollen as control. Then the DEGs between ‘Liuyefeitao’ and ‘Daifei’ at the early bloom stages by transcriptome sequencing were analyzed. The result showed that one S gene, and lots of non-S-locus factors such as: F-box proteins, Ub/26S, MAPKs, RLK, and transcription factor were differential expressed. We supposed that the strictly self-compatible of ‘Liuyefeitao’ may result from the synthesis of these S- locus and non-S-locus factors.

## Supporting information

S1 Table The primers of real-time PCR. (XLSX)

S2 Table Genes related to pollen recognition. (XLSX)
